# Integrated maps in quail (*Coturnix japonica*) confirm the high degree of synteny conservation with chicken (*Gallus gallus*) despite 35 million years of divergence

**DOI:** 10.1186/1471-2164-7-101

**Published:** 2006-05-02

**Authors:** Boniface B Kayang, Valérie Fillon, Miho Inoue-Murayama, Mitsuru Miwa, Sophie Leroux, Katia Fève, Jean-Louis Monvoisin, Frédérique Pitel, Matthieu Vignoles, Céline Mouilhayrat, Catherine Beaumont, Shin'ichi Ito, Francis Minvielle, Alain Vignal

**Affiliations:** 1 Laboratoire de Génétique Cellulaire, Centre INRA de Toulouse, BP 52627 Auzeville, 31326 Castanet Tolosan, France; 2 Faculty of Applied Biological Sciences, Gifu University, Gifu 501-1193, Japan; 3 UMR Génétique et Diversité Animales, INRA bât 211, 78352 Jouy-en-Josas Cedex, France; 4 Station de Recherches Avicoles, INRA, 37380 Nouzilly, France; 5 Department of Animal Science, University of Ghana, Legon, Accra, Ghana

## Abstract

**Background:**

By comparing the quail genome with that of chicken, chromosome rearrangements that have occurred in these two galliform species over 35 million years of evolution can be detected. From a more practical point of view, the definition of conserved syntenies helps to predict the position of genes in quail, based on information taken from the chicken sequence, thus enhancing the utility of this species in biological studies through a better knowledge of its genome structure. A microsatellite and an Amplified Fragment Length Polymorphism (AFLP) genetic map were previously published for quail, as well as comparative cytogenetic data with chicken for macrochromosomes. Quail genomics will benefit from the extension and the integration of these maps.

**Results:**

The integrated linkage map presented here is based on segregation analysis of both anonymous markers and functional gene loci in 1,050 quail from three independent F2 populations. Ninety-two loci are resolved into 14 autosomal linkage groups and a Z chromosome-specific linkage group, aligned with the quail AFLP map. The size of linkage groups ranges from 7.8 cM to 274.8 cM. The total map distance covers 904.3 cM with an average spacing of 9.7 cM between loci. The coverage is not complete, as macrochromosome CJA08, the gonosome CJAW and 23 microchromosomes have no marker assigned yet. Significant sequence identities of quail markers with chicken enabled the alignment of the quail linkage groups on the chicken genome sequence assembly. This, together with interspecific Fluorescence *In Situ *Hybridization (FISH), revealed very high similarities in marker order between the two species for the eight macrochromosomes and the 14 microchromosomes studied.

**Conclusion:**

Integrating the two microsatellite and the AFLP quail genetic maps greatly enhances the quality of the resulting information and will thus facilitate the identification of Quantitative Trait Loci (QTL). The alignment with the chicken chromosomes confirms the high conservation of gene order that was expected between the two species for macrochromosomes. By extending the comparative study to the microchromosomes, we suggest that a wealth of information can be mined in chicken, to be used for genome analyses in quail.

## Background

The Japanese quail (*Coturnix japonica*) is valued for its uniquely flavored eggs and meat and is reared in many countries of the world, particularly on a large scale in China, Japan, Brazil, Hong-Kong, France and Spain [1]. It is also an important animal model used in a range of scientific disciplines including embryonic development [2], behavior [3], physiology [4], genetics [5] and biomedicine [6]. In common with its close relative species the chicken, Japanese quail belongs to the the family Phasianidae in the order Galliformes and the two species have diverged 35 million years ago [7,8]. They have a karyotype of 2*n *= 78 chromosomes comprising a few morphologically distinct macrochromosomes (1–8 and the ZW sex chromosomes) and numerous cytologically indistinguishable microchromosomes. Moreover, chromosome homology between both species has been reported to be highly conserved, revealing only very few rearrangements [9]. This enables the nomenclature of the quail chromosomes (CJA for *Coturnix japonica*) to follow that of chicken by using corresponding numbers as suggested by the marker and gene data. However, unlike chicken where the majority of avian genomic studies have focused, much remains to be done on quail and other agriculturally and biologically important species. With the completion of the chicken genome map and sequence, a solid foundation has been laid on which comparative maps can be made for the less-studied poultry species. From this viewpoint, quail genome mapping would greatly profit from the unique relation between quail and chicken.

To further enhance the genetic improvement of this species as a food animal and also boost its potential as a research model for poultry, we have initiated mapping efforts in the Japanese quail, for which molecular information has been scarce until now. Indeed, mapping in quail has progressed from just three classical linkage groups based on plumage color and blood protein markers [10-13] to the first ever DNA-based genetic map constructed solely with AFLP markers [14] and to the recent microsatellite-based map [15]. However, both DNA-based maps were not only developed with different types of markers, but also used distinct populations. Therefore, to establish links between them, we genotyped markers from the microsatellite map in the population previously used for the AFLP map. Also, by adding a third mapping population, new microsatellite markers that were previously uninformative could be added to the integrated map.

Finally, to establish stronger links to the chicken maps and assembled sequence, we used three strategies: (i) gene loci were mapped in one population by developing Single Nucleotide Polymorphism (SNP) markers, (ii) microsatellite markers were located on the chicken sequence assembly by BLASTN searches, and (iii) comparative cytogenetic studies were conducted by means of FISH experiments.

## Results

Three mapping populations were used in the present study. Population 1 (Pop1) had previously been used to construct an AFLP map of quail and to map QTL for behavior traits [16]; population 2 (Pop2) to derive the first microsatellite map in quail and to map QTL [15,17]; and finally, population 3 (Pop3) to map plumage color and blood protein loci by microsatellite genotyping [18].

### Comprehensive microsatellite and gene maps

All the microsatellite markers available and informative were genotyped in Pop2 and Pop3, thus adding 14 markers to the previously published map. As the information on quail genes available in the public databases was scarce, to detect SNP in quail we opted to choose primers from chicken Expressed Sequence Tags (EST) with the understanding that the two species are closely related and, therefore, have highly conserved genes. All functional gene markers developed from chicken EST were tested by PCR for their suitability to amplify quail DNA, and the quail amplicon was sequenced in both forward and reverse directions to confirm orthology. Detection of polymorphism and genotyping of Type I markers were done only on Pop1 by Single Strand Conformation Polymorphism (SSCP) analysis. Therefore, to link together the microsatellite maps derived from Pop2 and Pop3 to the AFLP and gene-containing map derived from Pop1, a set of 63 microsatellite markers was chosen from the first microsatellite map [15] for genotyping Pop1. The choice of microsatellites was based on their position in the linkage group, so as to cover the entire map with a minimal set of markers to be used as anchor points.

**Figure 1:**
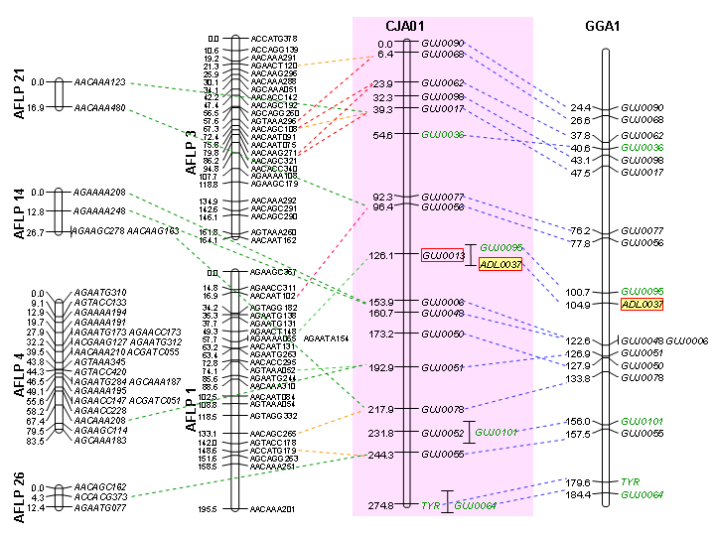
**Alignments of Japanese quail microsatellite and AFLP maps and links to the assembled chicken sequence**. Left: AFLP linkage groups from [14]; center: integrated microsatellite and gene map (this study); right: chicken February 2004 sequence assembly [22]. AFLP and microsatellite genetic maps are in cM and the sequence assembly is in Mb. New quail linkage groups have blue titles and new microsatellite and gene markers are in green. Genes are in boldface italic. Microsatellite markers informative in both species are in red boxes. Chicken markers have a yellow background. Quail microsatellites or genes with similarity to chicken sequence of unknown location (chrun) have a blue background. Dotted lines linking the microsatellite and the AFLP maps indicate positive results of two-point linkage between the markers, with supporting LOD scores of 3 to 6 (green), 6 to 10 (orange) or more than 10 (red).

**Figure 2:**
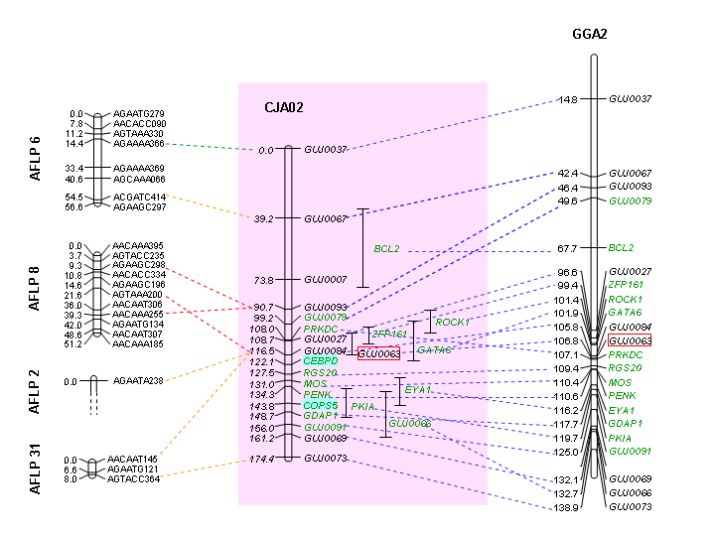
**Alignments of Japanese quail microsatellite and AFLP maps and links to the assembled chicken sequence**. Left: AFLP linkage groups from [14]; center: integrated microsatellite and gene map (this study); right: chicken February 2004 sequence assembly [22]. AFLP and microsatellite genetic maps are in cM and the sequence assembly is in Mb. New quail linkage groups have blue titles and new microsatellite and gene markers are in green. Genes are in boldface italic. Microsatellite markers informative in both species are in red boxes. Chicken markers have a yellow background. Quail microsatellites or genes with similarity to chicken sequence of unknown location (chrun) have a blue background. Dotted lines linking the microsatellite and the AFLP maps indicate positive results of two-point linkage between the markers, with supporting LOD scores of 3 to 6 (green), 6 to 10 (orange) or more than 10 (red).

**Figure 3:**
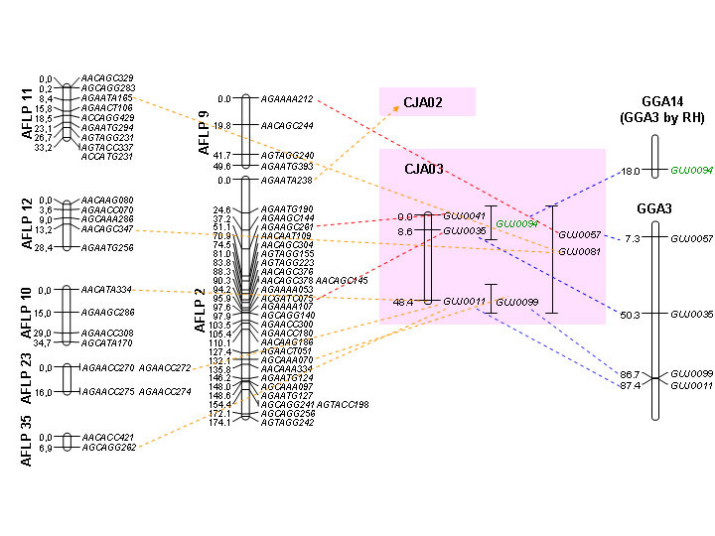
**Alignments of Japanese quail microsatellite and AFLP maps and links to the assembled chicken sequence**. Left: AFLP linkage groups from [14]; center: integrated microsatellite and gene map (this study); right: chicken February 2004 sequence assembly [22]. AFLP and microsatellite genetic maps are in cM and the sequence assembly is in Mb. New quail linkage groups have blue titles and new microsatellite and gene markers are in green. Genes are in boldface italic. Microsatellite markers informative in both species are in red boxes. Chicken markers have a yellow background. Quail microsatellites or genes with similarity to chicken sequence of unknown location (chrun) have a blue background. Dotted lines linking the microsatellite and the AFLP maps indicate positive results of two-point linkage between the markers, with supporting LOD scores of 3 to 6 (green), 6 to 10 (orange) or more than 10 (red).

**Figure 4:**
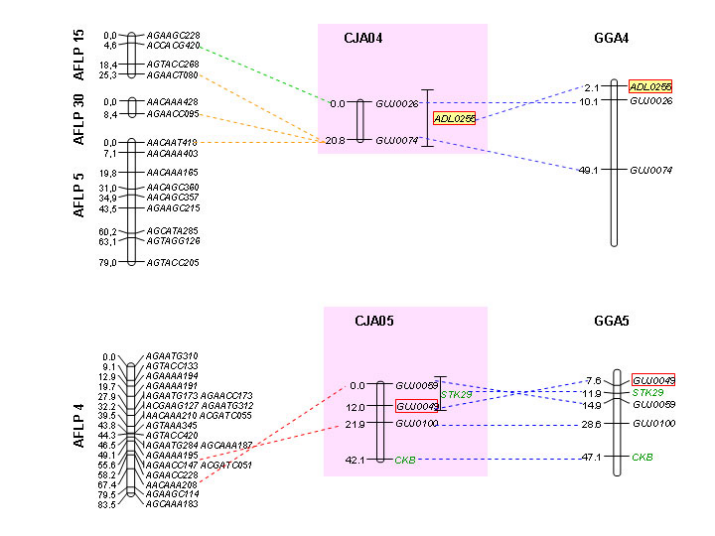
**Alignments of Japanese quail microsatellite and AFLP maps and links to the assembled chicken sequence**. Left: AFLP linkage groups from [14]; center: integrated microsatellite and gene map (this study); right: chicken February 2004 sequence assembly [22]. AFLP and microsatellite genetic maps are in cM and the sequence assembly is in Mb. New quail linkage groups have blue titles and new microsatellite and gene markers are in green. Genes are in boldface italic. Microsatellite markers informative in both species are in red boxes. Chicken markers have a yellow background. Quail microsatellites or genes with similarity to chicken sequence of unknown location (chrun) have a blue background. Dotted lines linking the microsatellite and the AFLP maps indicate positive results of two-point linkage between the markers, with supporting LOD scores of 3 to 6 (green), 6 to 10 (orange) or more than 10 (red).

**Figure 5:**
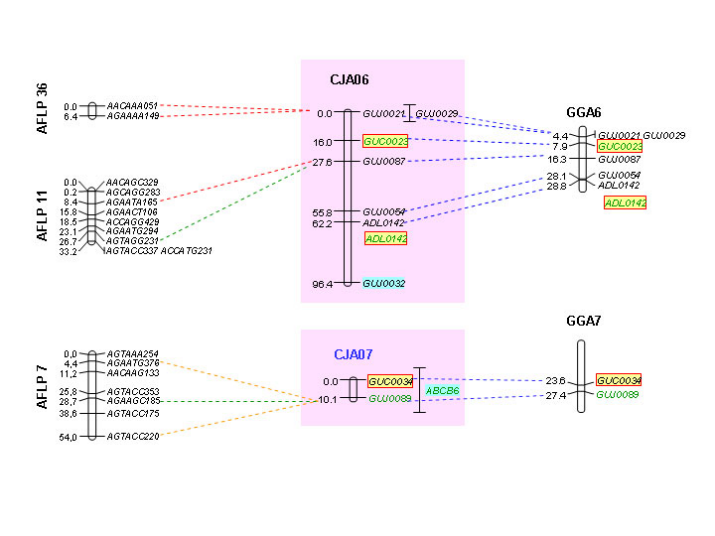
**Alignments of Japanese quail microsatellite and AFLP maps and links to the assembled chicken sequence**. Left: AFLP linkage groups from [14]; center: integrated microsatellite and gene map (this study); right: chicken February 2004 sequence assembly [22]. AFLP and microsatellite genetic maps are in cM and the sequence assembly is in Mb. New quail linkage groups have blue titles and new microsatellite and gene markers are in green. Genes are in boldface italic. Microsatellite markers informative in both species are in red boxes. Chicken markers have a yellow background. Quail microsatellites or genes with similarity to chicken sequence of unknown location (chrun) have a blue background. Dotted lines linking the microsatellite and the AFLP maps indicate positive results of two-point linkage between the markers, with supporting LOD scores of 3 to 6 (green), 6 to 10 (orange) or more than 10 (red).

**Figure 6:**
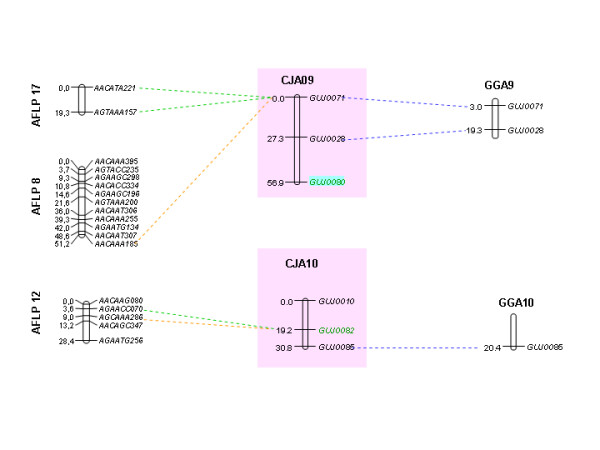
**Alignments of Japanese quail microsatellite and AFLP maps and links to the assembled chicken sequence**. Left: AFLP linkage groups from [14]; center: integrated microsatellite and gene map (this study); right: chicken February 2004 sequence assembly [22]. AFLP and microsatellite genetic maps are in cM and the sequence assembly is in Mb. New quail linkage groups have blue titles and new microsatellite and gene markers are in green. Genes are in boldface italic. Microsatellite markers informative in both species are in red boxes. Chicken markers have a yellow background. Quail microsatellites or genes with similarity to chicken sequence of unknown location (chrun) have a blue background. Dotted lines linking the microsatellite and the AFLP maps indicate positive results of two-point linkage between the markers, with supporting LOD scores of 3 to 6 (green), 6 to 10 (orange) or more than 10 (red).

**Figure 7:**
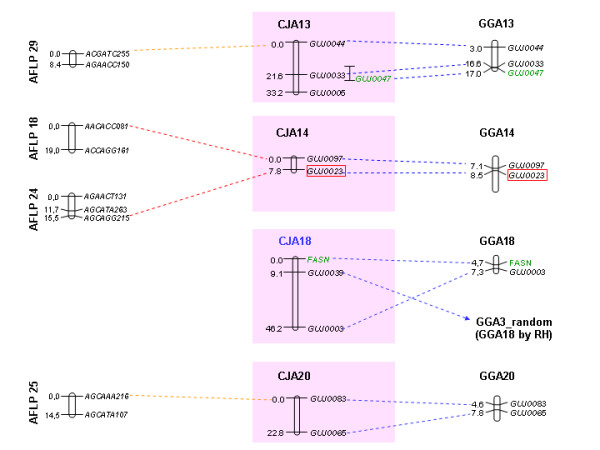
**Alignments of Japanese quail microsatellite and AFLP maps and links to the assembled chicken sequence**. Left: AFLP linkage groups from [14]; center: integrated microsatellite and gene map (this study); right: chicken February 2004 sequence assembly [22]. AFLP and microsatellite genetic maps are in cM and the sequence assembly is in Mb. New quail linkage groups have blue titles and new microsatellite and gene markers are in green. Genes are in boldface italic. Microsatellite markers informative in both species are in red boxes. Chicken markers have a yellow background. Quail microsatellites or genes with similarity to chicken sequence of unknown location (chrun) have a blue background. Dotted lines linking the microsatellite and the AFLP maps indicate positive results of two-point linkage between the markers, with supporting LOD scores of 3 to 6 (green), 6 to 10 (orange) or more than 10 (red).

**Figure 8:**
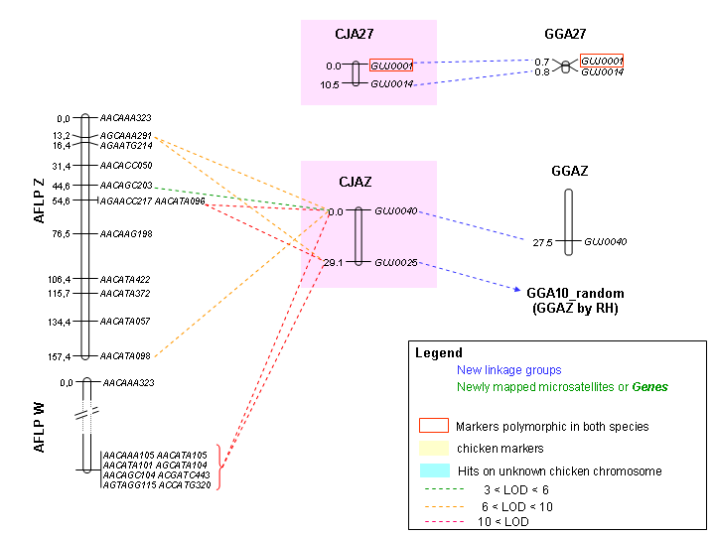
**Alignments of Japanese quail microsatellite and AFLP maps and links to the assembled chicken sequence**. Left: AFLP linkage groups from [14]; center: integrated microsatellite and gene map (this study); right: chicken February 2004 sequence assembly [22]. AFLP and microsatellite genetic maps are in cM and the sequence assembly is in Mb. New quail linkage groups have blue titles and new microsatellite and gene markers are in green. Genes are in boldface italic. Microsatellite markers informative in both species are in red boxes. Chicken markers have a yellow background. Quail microsatellites or genes with similarity to chicken sequence of unknown location (chrun) have a blue background. Dotted lines linking the microsatellite and the AFLP maps indicate positive results of two-point linkage between the markers, with supporting LOD scores of 3 to 6 (green), 6 to 10 (orange) or more than 10 (red).

In all, 112 markers comprising 90 microsatellites and 22 functional genes were analyzed by two-point linkage analysis including the genotype data from the three populations. The number of informative meioses in the comprehensive F2 mapping population of 1,050 quail varied from 42 to 1,618 with an average of 726 per locus and was therefore sufficient to assure high levels of support for ordering the linkage groups. Ninety-two of the markers, representing 74 microsatellites and 18 genes, were resolved into 14 autosomal linkage groups and a Z chromosome-specific linkage group (Figure 1 to 8) while the remaining 20 markers (16 microsatellites and four genes) showed no linkage to any other marker. The size of the linkage groups ranges from 7.8 cM (CJA14) to 274.8 cM (CJA01) and the overall map coverage within linkage groups is 904.3 cM with an average spacing of 9.7 cM between loci. Thus, the integration of data from the three mapping populations and the inclusion of additional markers has enabled the extension of the microsatellite and gene-containing linkage groups from 576 cM [15] to 904 cM. Most of the functional gene markers were assigned to CJA02 since they were developed for chromosome 2 as part of a study to confirm a putative QTL.

### Aligning the comprehensive map to the AFLP map

After microsatellite genotyping in Pop1, two-point linkage analysis between these new markers and the AFLP was used to establish the links with the maps derived from Pop2 and Pop3. Overall, by using a LOD score threshold of 3.0, 31 out of the 41 AFLP linkage groups were linked to 13 out of the 15 microsatellite linkage groups (Figure 1 to 8). The remaining 10 AFLP linkage groups were amongst the smallest. This suggests that neither map has a complete coverage of the genome. CJA01 and CJA03 had links to as many as six and seven AFLP groups, respectively (Figure 1 to 8). Generally, large AFLP linkage groups were associated with linkage groups assigned to macrochromosomes (e.g. CJA01 – CJA07) while small AFLP groups were linked to integrated linkage groups assigned to microchromosomes (e.g. CJA09 – CJA27). Both the Z and W AFLP groups, however, were linked to the Z-linked group CJAZ. In some instances, one AFLP linkage group is linked to two different microsatellite linkage groups. For example, the terminal marker of AFLP2 is linked to CJA02, whereas the rest of AFLP2 is linked to CJA03; likewise, AFLP8 has markers linked to CJA02 and CJA09.

### Alignment with the chicken sequence

A BLASTN search of all the microsatellites on the quail map against the available chicken sequence assembly yielded significant hits for 61 out of 69 (88.4%) of the quail-originated markers (Table 1) [see Additional file 1]. The remaining 8 markers had no hits. Sequence identities ranged from 63.6% to 100%, alignment scores from 158 to 848, and E-values from 43.1E-31 to 2.8. For a number of markers, a microsatellite, though sometimes interrupted or very short, could be found in the chicken sequence. All the Type I markers, except *ABCB6*, which had no hit to the chicken genome sequence, produced significant hits, with E-values ranging from 8.90E-247 to 2.40E-17 (Table 2) [see Additional file 2]. These values reflect the high degree of sequence conservation between chicken and quail, especially for Type I markers. Although *ABCB6 *had no direct hit to the chicken genome assembly, it matches the chicken EST BI391579, which aligns with the genome assembly in the fraction of sequences not attributed to a chromosome (chrun) at 101.609 Mb.

A plot of the sequence coordinates of both Type I and Type II markers on the chicken sequence assembly enabled the alignments of the integrated quail maps with chicken chromosomes, thus permitting the correct orientation and assignment of all the quail linkage groups (Figure 1 to 8). These alignments revealed remarkable similarity in the order of markers between chicken and quail in both the macrochromosomes (CJA01-07) and the microchromosomes (CJA09, 10, 13, 14, 18, 20, 27).

Inconsistent marker order between the two species suggestive of local chromosomal inversions or of errors in one or the other dataset was observed on chromosomes 1, 2 and 5. Linkage mapping has a lower resolution than the sequence assembly, but the latter may have portions of chromosomes in wrong places. It is therefore difficult to make a decision on marker order, especially as these rearrangements can be real local inversions.

All the markers present together on any of the quail linkage groups went together on a single chicken chromosome, with three notable exceptions in CJA03, CJA18 and CJAZ. The marker *GUJ0094 *from linkage group CJA03 indicated strong sequence similarity to GGA14 sequence at position 18 Mb. However, a comparative study between a RH map and the sequence assembly of GGA14 showed that the portion between 17 and 19.5 Mb of the latter corresponds, in fact, to GGA3 [19]. In the case of CJA18 and CJAZ, the markers *GUJ0039 *and *GUJ0025 *showed strong sequence similarity to portions of GGA3_random and GGA10_random, respectively. Considering that these markers were assigned to "improperly" assembled portions of the chromosomes designated as "random", we decided to investigate these discrepancies. Both *GUJ0039 *and *GUJ0025 *were genotyped on the ChickRH6 panel [20] and were found by two-point linkage analysis to co-segregate with markers belonging to GGA18 and GGAZ, respectively [21]. Thus, when correcting chicken sequence assembly errors by RH mapping, we find that all markers from a given quail linkage group correspond to one chicken chromosome.

### Comparative FISH studies

**Figure 9:**
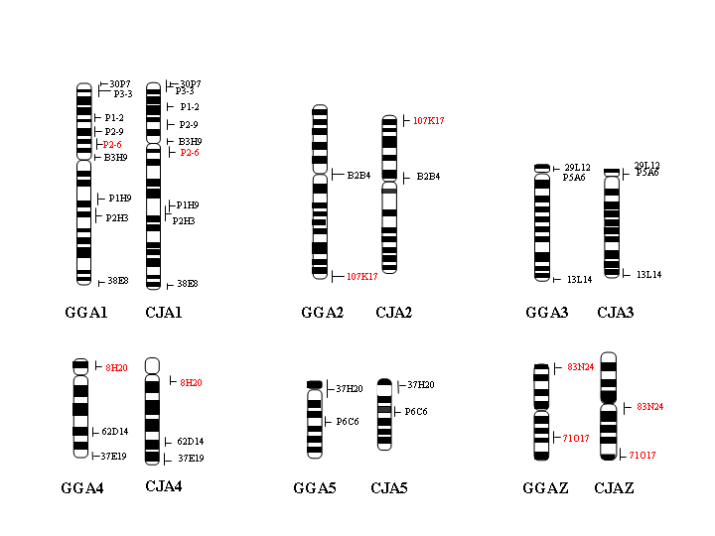
**FISH of chicken macrochromosome BAC clones to quail chromosomes**. Standard chicken and quail G-banding ideograms are from [52]. Markers mapped to different positions in both species are in red. Results on smaller macrochromosomes and microchromosomes are reported in Table 3.

All 49 chicken BAC clones tested for hybridization to quail metaphase chromosomes gave a positive FISH signal. The 29 clones from chicken macrochromosomes 1–8 and Z were found in almost the same order on their quail counterparts and only few differences suggesting centromeric inversions in chromosomes 1, 2 and 4 could be detected (Table 3 and Figure 9). For chromosome 1, the markers P2-6 and B3H9 are both located on the p arm in chicken whereas in quail their positions are reversed: P2-6 is located on the q arm close to the centromere and B3H9 is found in the centromeric region. In chromosome 2, the markers B2B4 and bw107K17 are inverted between chicken and quail, while in chromosome 4 the marker bw8H20 on the p arm in chicken appears on the q arm in quail. All 20 BAC clones from chicken microchromosomes hybridized to microchromosomes in quail. For seven chicken microchromosomes, two clones were chosen as far as possible from one another on the corresponding genetic linkage groups, so as to ensure a good coverage. Such pairs of clones were analyzed simultaneously in dual-color FISH experiments. In all cases, clones co-hybridized to the same quail microchromosome, suggesting high conservation between both species. Altogether, 13 chicken microchromosomes were investigated, allowing identification of their quail counterparts.

## Discussion

### Integrated maps for QTL studies

To date, this is the most enhanced genetic linkage map presented for the Japanese quail. It is an improvement over the microsatellite linkage map of Kayang *et al*. (2004) [15], both in terms of marker number and quality, with a number of Type I loci added, and a wider coverage of the quail genome. An added value is the alignments with the AFLP linkage map of Roussot *et al*. (2003) [14], thus permitting the chromosomal assignment of 31 out of the 41 AFLP linkage groups. Neither of the two maps looks complete and the microsatellite map has a lower number of linkage groups than chromosomes, due to the difficulty in mapping the small microchromosomes. However, the microsatellite map appears to be a great improvement over the AFLP map, in which even the macrochromosomes appear to be fragmented into several linkage groups and some AFLP markers seem to be assigned to the wrong linkage group. This demonstrates the limits of using AFLP markers in a F2 cross between two lines that are only partially inbred. Nevertheless, the alignment of both maps is useful as it enables a comparison of the location of QTL found in the different crosses. For instance, the QTL on tonic immobility found on the AFLP linkage group 1 [16] can now be related to the suggestive QTL for the same trait found on CJA01 [17].

The majority of poultry (and indeed avian) genomic studies have focused on the chicken, culminating in the sequencing of its entire genome [22]. The slow pace of mapping in other poultry genomes primarily can be attributed to the paucity of markers, particularly microsatellites, which are the markers of choice for genetic mapping and QTL detection, and also to the fact that only a few groups work on species other than chicken. Microsatellite markers are indeed difficult to develop in large numbers for poultry because of a relatively lower frequency of occurrence, when compared to mammals [23]. Chicken microsatellite markers could be a source for the development of the quail map, but there is a low success rate of cross-species amplification between the two species [24,25]. Studies by Primmer *et al*, (1996) [26] in passerine birds revealed a significant negative correlation between microsatellite performance and evolutionary distance between species, with 50% of the markers revealing polymorphism if the divergence between the original and the tested species is 11 million years. Thus, the success rate of chicken microsatellites in quail is expected to be lower, due to a divergence that goes back to 35 million years [7,8]. An alternative to microsatellites for genetic mapping is to use SNP. Our results demonstrate that the use of information on the sequence, the structure and the position of genes in the genome of chicken, is a good approach for a targeted development of SNP in a defined region in quail, in our case CJA02.

Further compounding the situation in birds is the numerous indistinguishable microchromosomes. Thus, apart from the Japanese quail, preliminary genetic linkage maps have only been reported for the turkey [27,28] but are yet to be published for most important poultry species. Given the difficulty in mapping avian genomes, this integrated map is a further boost to genomic studies in quail and is a valuable resource that will improve the localization of QTL for the commercial improvement of this species and will help its promotion as a laboratory model for poultry.

### Chromosome rearrangements since the divergence of quail and chicken

Altogether, the genetic and FISH comparative maps revealed very high similarities in marker order between the two species both for the macrochromosome (CJA01-08, CJAZ) and the microchromosome (CJA09, 10, 11, 13, 14, 15, 17, 18, 19, 20, 24, 26, 27, 28) fractions of the genome. Therefore, all the syntenic segments investigated were conserved.

Several studies suggest a high stability of avian karyotypes, which is now well documented for macrochromosomes. The karyotype of most bird orders usually share characteristic features, such as relatively high chromosome numbers (76 ≤ 2*n *≤ 84), the presence of few macrochromosomes and numerous morphologically indistinguishable microchromosomes, and ZZ/ZW sex chromosomes. Comparative analyses of macrochromosomes for several bird species by using chicken whole chromosome painting probes in cross-species experiments have shown a low degree of inter-chromosomal rearrangements [29,30]. The high degree of sequence identity and similarities in marker order revealed by the comparative mapping with chicken performed in the present study demonstrates that the intra-chromosomic rearrangements are also rare. However, it is not impossible that some of the differences found in marker order between the quail and chicken maps presented here may be due to the lack of precision in marker ordering on the quail genetic map or errors in the chicken genome sequence assembly. The cytogenetic data shows that the rearrangements on chromosomes 1, 2 and 4 all involve a change in the position of the centromere (Figure 9), thereby confirming similar findings by Shibusawa *et al*. (2001) [9]. Until now, all FISH studies on microchromosomes proved a strong conservation of synteny, with hybridization to chromosomes of similar sizes. For all 23 chromosomes investigated, no interchromosomal rearrangements could be detected. Although it is difficult to estimate the degree of coverage of microchromosomes in our study, for seven of the microchromosomes, BAC clones were chosen from the ends of the chicken genetic linkage group, to maximize the chances of detecting interchromosomal rearrangements. The time of divergence between chicken and quail is estimated to be 35 million years [7,8]. Similar times of divergence can be found between humans and New World monkeys (35 million years) or humans and Old World monkeys (30 million years) [31], for which numerous interchromosomal events have been documented [32,33]. This suggests a higher stability of karyotypes in galliformes than in primates.

### Genome coverage by the microsatellite map

An important point when building genetic maps is to estimate the degree of genome coverage within the linkage groups. As described above, the integrated map comprises 15 linkage groups spanning 904.3 cM and 20 unlinked markers. CJA08, CJAW and 23 microchromosomes are still missing. It is quite probable that the unlinked markers belong to these chromosomes. This is not surprising as even in chicken, the genetic map and the sequence assembly for a number of microchromosomes are far from being complete. Assuming 50 cM for each unlinked marker, the total map distance would be 1,904 cM. If the genome size of quail is comparable to that of chicken (3,800 cM), then the comprehensive map represents 50% of the quail genome. Indeed, apart from chromosome 8, all the macrochromosomes are represented and the marker coverage and density for chromosomes 1 and 2 in particular, are high enough to permit the localization of QTL. Moreover, the presence of functional genes in the linkage groups, especially in CJA02, would be useful for comparative mapping and evolutionary studies.

## Conclusion

We have presented here the most comprehensive quail map to date, obtained by aligning all microsatellite data with the AFLP map and comparing it with the chicken sequence. These results together with our FISH experiments confirm the high level of synteny conservation between the two species. The availability of a comparative map between quail and chicken will accelerate mapping studies in quail not only by facilitating the transfer of genetic information in the form of markers but also gene predictions directly from chicken to quail. The level of detail is sufficient to allow the mapping of QTL, but subsequent efforts should aim at covering the missing parts of the genome by taking advantage of the sequenced chicken genome. Also, further integration of data and markers from one other group [34] should be pursued.

## Methods

### Quail resource populations

Three Japanese quail populations were used in this study: two established at INRA experimental units in Nouzilly, France and one developed at Gifu University, Gifu, Japan. Pop1 was derived from two quail lines of the same genetic origin that had been divergently selected for short (STI) or long (LTI) duration of tonic immobility, a fear-related behavior trait [16]. Pop2 was derived from two quail lines of different genetic origins: line LTI selected for fearfulness and line DD selected for early egg production [35]. Pop3 consisted of 193 F2 individuals from 25 full-sib families derived from crosses between the wild-type and four plumage color mutant lines: silver (*B*), black at hatch (*Bh*), extended brown (*E*), and yellow (*Y*). Ten of these families also carried the celadon eggshell color mutation (*ce*) [36]. The comprehensive map was, therefore, based on the genotyping results of a total of 1,050 quail from the three resource populations.

### Microsatellite (Type II) markers

Most microsatellite markers used in this study were of quail origin. These included 82 previously published markers [37,38] and two new ones: *GUJ0101 *(GAGTGATAGGCTGAGAAAAC ; GCTTACCTATGTTCAGCTTG) and *GUJ0102 *(CTGGTAACTTCTTGCAGCCA ; GCTATAAGAAAGCACGGGAG) with respective GenBank accession numbers AB181537 and AB181538. In addition, six chicken markers (*ADL0037*, *ADL0142*, *ADL0255*, *GUC0023*, *GUC0028*, and *GUC0034*) that cross-amplify quail DNA, reveal several alleles and are orthologous to the quail loci, were also included.

### Functional gene (Type I) marker development

Most of the primers for Type I markers (Table 2) were chosen from chicken EST data aligned to the human sequence using the ICCARE (Interspecific Comparative Clustering and Annotation foR EST) web server [39]. This strategy enables one to predict the position of markers in chicken, through known synteny conservation data with human. Primers were chosen in exons for increasing cross-species PCR success rate, taking care to include small introns for maximizing chances of finding SNP. Finally, primer sequences were designed using the PRIMER3 server [40].

PCR of microsatellite markers were carried out in Pop2 as previously described [15]. In Pop1, PCR amplifications were carried out in 10 μl reaction mixtures containing 15 ng of genomic DNA, 0.3 μM of forward and reverse primers, 100 μM of each dNTP, 1.5 to 2.5 mM MgCl_2 _as determined by test experiments, 0.5 U *Taq *polymerase (Invitrogen Life Technologies, CA, USA) and 1 X buffer (Invitrogen). The reverse primers were pre-labelled with fluorescent dyes: 6-FAM, HEX or NED fluorophores for automated genotyping. After an initial incubation for 5 min at 94°C, 30 PCR cycles of 30 sec at 94°C, 30 sec at annealing temperature and 30 sec at 72°C were performed, with a final elongation step at 72°C for 10 min.

PCR amplifications of gene markers were performed in 25 μl reaction mixtures containing 25 ng of genomic DNA, 0.2 μM of forward and reverse primers, 200 μM of each dNTP, 2 mM MgCl_2_, 0.5 U *Taq *DNA polymerase (Invitrogen) and 1 X Invitrogen PCR buffer. The PCR cycling conditions were identical to those used for the microsatellite markers. PCR product sizes were determined by electrophoresis on 2% agarose gels in 1 X Tris-Borate-EDTA (TBE) buffer for 30 min at 200 V in the presence of 5 μl of Smart Ladder DNA size standard (Eurogentec, Seraing, Belgium). The gels were stained with ethidium bromide and visualized under UV light.

### Sequencing

Quail gene marker PCR products were purified with the QIAquick PCR Purification Kit (Qiagen, Valencia, CA, USA) and cycle sequencing reactions were performed in 10 μl with 10 to 20 ng DNA, 10 pmol of one of the PCR primers, and 2 μl of Big Dye Terminator Mix (Applied Biosystems, Foster City, CA, USA). After an initial denaturation at 94°C for 5 min, 25 PCR cycles of 30 sec at 94°C, 15 sec at the annealing temperature and 4 min at 60°C were carried out. The products were then purified with the QIAquick Kit (Quiagen) and analyzed on an ABI Prism 3700 DNA Sequencer (Applied Biosystems). The identity of each gene was checked by sequence comparison with the chicken EST.

### Genotyping

Microsatellite PCR products were combined into sets of 3 to 8 markers according to size and dye color compatibility, electrophoresed on ABI Prism 3100 or 3700 DNA Sequencers (Applied Biosystems) and sized using Genescan version 3.7 software (Applied Biosystems). Genotyping analysis was performed using Genotyper version 3.7 software (Applied Biosystems).

Detection of polymorphism and genotyping of Type I markers were done on Pop1 by SSCP analysis. PCR products were denatured at 94°C for 5 min and loaded on a 10% polyacrylamide gel (acrylamide: bisacrylamide 49:1), containing 5% glycerol. Electrophoresis was run at 15°C between 400 and 700 V for 14 to 24 h depending on the size of the fragment being analyzed. The results were visualized by AgNO_3 _staining [41]. Each marker was first tested on the F1 individuals of Pop1 to detect polymorphism before genotyping the whole population.

### Linkage analysis and map construction

Linkage analysis was performed using CriMap version 2.4 software [42]. Correct Mendelian inheritance of marker alleles was checked with the PREPARE option. Discordant data was rechecked within Genotyper and if no correction was possible, individuals were retyped. A two-point linkage analysis of all the markers was then computed using a LOD score threshold of 3.0 to assign markers to linkage groups. Subsequently, the BUILD option was used to order markers within each linkage group. Two or three loci with the highest number of informative meioses were chosen as the ordered loci and additional markers in the group were sequentially incorporated one by one at every possible location. At each step, the likelihood of the order was computed and the order with the maximum likelihood was retained. The process ended when no more markers could be inserted. Finally, the FLIPS option was used to examine the order of the different loci within each linkage group by inverting every two or three loci, thus verifying the robustness of the linkage groups and eventually finding the best order. Map distances were derived based on the Kosambi mapping function and the maps were drawn using MapChart version 2.0 [43].

### FISH under heterologous conditions

Large-insert clones for FISH mapping were from two different BAC libraries and one PAC library [44,45]. Clones for heterologous FISH mapping in quail were selected according to their known position in chicken, as determined by FISH or by genetic mapping of a microsatellite or SSCP marker [46-48].

Quail metaphase spreads were obtained from seven-day old embryo fibroblast cultures arrested 4 hours with 0.06 μg/ml colcemid (Gibco BRL, Grand Island, NY) and fixed by standard procedures.

For single color FISH, 100 ng of clone DNA were labelled by random priming using biotin-16-dUTP (Boehringer-Mannheim, Mannheim, Germany). Probes were then hybridized *in situ *for 48 hours in a hybridization buffer containing 30% formamide, after which the slides were washed in 40% formamide at 42°C, followed by detections with avidin-fluorescein isothiocyanate (FITC) (Vector Laboratories Inc., Burlingame, CA). Chromosomes were counterstained with propidium iodide (Sigma Chemical Co., St Louis, MO) in antifade solution (Vector) [49].

Two-color FISH was performed using a total of 20 chicken clones for localization on quail microchromosomes (Table 3). Labelling was done with digoxigenin (digoxigenin-11-dUTP, Boehringer-Mannheim) or biotin (biotin-16-dUTP, Boehringer-Mannheim). Biotin-labelled probes were detected with avidin-texas red (Vector) and digoxigenin-labelled ones with FITC antibodies (Boehringer-Mannheim). Chromosomes were counterstained with DAPI (4', 6-diamidino-2-phenilindole-dihydrochloride, Sigma) in antifade solution (Vector) [50].

The hybridized metaphases were screened with a Zeiss fluorescence microscope and a minimum of twenty spreads was analyzed for each experiment. Spot-bearing metaphases were captured and analyzed with a cooled CCD camera using Cytovision software (Applied Imaging, Sunderland, UK).

### Alignment of the Japanese quail map with the chicken sequence

The Ensembl Genome Browser [51] was used to perform BLASTN searches of all the markers on the quail linkage groups against the assembled chicken sequence (Table 1). Linkage and sequence maps were drawn using the MapChart program [43].

## Authors' contributions

BBK and KF carried out the genotyping. BBK performed the genetic mapping work and drafted the manuscript. VF and MV carried out the FISH analyses. SL, CM and FP developed markers and produced genotypes for Type I markers. CB supervised the production of quail population 1, FM and J-LM of population 2 and MIM, MMi and SI of population 3. AV coordinated the study and finalized the manuscript.

## Supplementary Material

Additional File 1 **Top BLASTN hits of Japanese quail microsatellite markers on the chicken genomic sequence**. This table describes details on the BLASTN hits of the quail microsatellite markers on the chicken genome: chicken chromosome, position in bp, % similarity, lengths of the alignment, alignment score and e-value. When microsatellites were found in the chicken sequence, they were reported. Click here for file

Additional File 2 **Top BLASTN hits of Japanese quail gene markers on the chicken genomic sequence**. This table describes details on the BLASTN hits of the quail gene fragment sequences on the chicken genome: chicken chromosome, position in bp, % similarity, lengths of the alignment, alignment score and e-value. Click here for file

## Figures and Tables

**Table 1:** Location of Japanese quail microsatellite markers from the genetic map on the chicken February 2004 genome assembly by BLASTN.

Quail locus^1^	Origin	Linkage group	Position (cM)	Chicken Chr:Mb^3^	E-value^4^
*GUJ0090*	quail	CJA01	0	chr1:24.420	2.3E-01
*GUJ0068*	quail	CJA01	6	chr1:26.583	2.9E-26
*GUJ0062*	quail	CJA01	24	chr1:37.771	2.0E-09
*GUJ0098*	quail	CJA01	32	chr1:43.072	2.5E-24
*GUJ0017*	quail	CJA01	39	chr1:47.501	2.5E-17
*GUJ0036*^2^	quail	CJA01	55	chr1:40.563	1.1E-11
*GUJ0077*	quail	CJA01	92	chr1:76.184	6.1E-28
*GUJ0056*	quail	CJA01	96	chr1:77.751	1.2E-05
*GUJ0013*	quail	CJA01	126	no hit	-
*GUJ0006*	quail	CJA01	154	chr1:122.564	4.9E-03
*GUJ0048*	quail	CJA01	161	chr1:122.564	1.7E-05
*GUJ0050*	quail	CJA01	173	chr1:127.921	1.1E-09
*GUJ0051*	quail	CJA01	193	chr1:126.894	3.5E-10
*GUJ0078*	quail	CJA01	218	chr1:133.776	2.0E-01
*GUJ0052*	quail	CJA01	232	no hit	-
*GUJ0055*	quail	CJA01	244	chr1:157.520	1.8E-06
*GUJ0101*	quail	CJA01	± 232	chr1:156.040	2.1E-16
*ADL0037*	chicken	CJA01	± 126	chr1:104.907	3.2E-50
*GUJ0095*^2^	quail	CJA01	± 126	chr1:100.717	1.4E-02
*GUJ0064*^2^	quail	CJA01	± 275	chr1:184.395	6.6E-23
*GUJ0037*	quail	CJA02	0	chr2:14.772	1.3E-18
*GUJ0067*	quail	CJA02	39	chr2:42.387	4.4E-04
*GUJ0007*	quail	CJA02	74	no hit	-
*GUJ0093*	quail	CJA02	91	chr2:46.413	2.3E-03
*GUJ0079*^2^	quail	CJA02	99	chr2:49.645	1.2E-01
*GUJ0027*	quail	CJA02	109	chr2:96.645	2.7E-09
*GUJ0084*	quail	CJA02	117	chr2:105.833	4.5E-06
*GUJ0091*^2^	quail	CJA02	156	chr2:125.035	9.2E-14
*GUJ0069*	quail	CJA02	161	chr2:132.081	1.1E-19
*GUJ0073*	quail	CJA02	174	chr2:138.921	8.3E-07
*GUJ0063*	quail	CJA02	± 110	chr2:106.842	4.9E-26
*GUJ0066*^2^	quail	CJA02	± 150	chr2:132.662	1.2E-07
*GUJ0041*	quail	CJA03	0	no hit	-
*GUJ0035*	quail	CJA03	9	chr3:50.291	3.5E-09
*GUJ0011*	quail	CJA03	48	chr3:87.368	3.0E-05
*GUJ0057*	quail	CJA03	± 20	chr3:7.343	5.6E-11
*GUJ0081*	quail	CJA03	± 20	no hit	-
*GUJ0099*	quail	CJA03	± 48	chr3:86.666	7.6E-25
*GUJ0094*^2^	quail	CJA03	± 5	chr14:18.036	2.9E-22
*GUJ0026*	quail	CJA04	0	chr4:10.067	2.8E+00
*GUJ0074*	quail	CJA04	21	chr4:49.116	3.1E-31
*ADL0255*^2^	chicken	CJA04	± 10	chr4:2.089	4.2E-54
*GUJ0059*	quail	CJA05	0	chr5:14.850	3.2E-25
*GUJ0049*	quail	CJA05	12	chr5:7.574	1.9E-21
*GUJ0100*	quail	CJA05	22	chr5:28.592	1.6E-16
*GUJ0021*	quail	CJA06	0	chr6:4.398	1.9E-13
*GUC0023*^2^	chicken	CJA06	16	chr6:7.890	2.4E-25
*GUJ0087*	quail	CJA06	28	chr6:16.277	1.2E-11
*GUJ0054*	quail	CJA06	56	chr6:28.056	5.6E-07
*ADL0142*	chicken	CJA06	62	chr6:28.806	1.2E-40
*GUJ0032*^2^	quail	CJA06	96	chrun:41.057	1.4E-04
*GUJ0029*	quail	CJA06	± 0	chr6:4.398	2.4E-12
*GUC0034*^2^	chicken	CJA07	0	chr7:23.564	6.5E-11
*GUJ0089*^2^	quail	CJA07	10	chr7:27.428	1.7E-05
*GUJ0071*	quail	CJA09	0	chr9:3.000	8.4E-13
*GUJ0028*	quail	CJA09	27	chr9:19.281	2.5E-11
*GUJ0080*^2^	quail	CJA09	57	chrun:0.025	1.1E+00
*GUJ0010*	quail	CJA10	0	no hit	-
*GUJ0082*^2^	quail	CJA10	19	no hit	-
*GUJ0085*	quail	CJA10	31	chr10:20.429	1.6E-19
*GUJ0044*	quail	CJA13	0	chr13:3.033	6.7E-16
*GUJ0033*	quail	CJA13	22	chr13:16.646	1.0E-11
*GUJ0005*	quail	CJA13	33	no hit	-
*GUJ0047*^2^	quail	CJA13	± 22	chr13:17.033	5.5E-28
*GUJ0097*	quail	CJA14	0	chr14:7.106	5.7E-09
*GUJ0023*	quail	CJA14	8	chr14:8.487	4.4E-15
*GUJ0039*	quail	CJA18	9	chr3_random	2.6E-13
*GUJ0003*	quail	CJA18	46	chr18:7.342	1.3E-11
*GUJ0083*	quail	CJA20	0	chr20:4.643	2.6E-02
*GUJ0065*	quail	CJA20	23	chr20:7.845	7.9E-06
*GUJ0001*	quail	CJA27	0	chr27:0.677	4.5E-25
*GUJ0014*	quail	CJA27	11	chr27:0.750	1.5E-07
*GUJ0040*	quail	CJAZ	0	chrZ:27.527	1.3E-16
*GUJ0025*	quail	CJAZ	29	chr10_random	2.4E-21

^1^*GUJ *markers were developed by Kayang *et al*. (2000, 2002) [37, 38], *GUC *markers were developed by Kayang *et al *(2003) [53], *ADL *markers were developed by Cheng and Crittenden (1994) [54]; Cheng *et al*. (1995) [55].

^2^Newly mapped microsatellites

^3^Position of the quail microsatellite sequence on the chicken genome sequence assembly, determined by BLASTN

^4^E-value of the quail-chicken alignment

± indicates the median position of non-framework markers Chrun: chicken genomic sequence of unknown location. Chr_random: chicken genomic sequence assigned to, but not located on a chromosome.

**Table 2:** Location by BLASTN of quail genetic map Type I markers on the chicken genome assembly

Marker	Gene^1^	Chicken chr.^2^	Fragment Position^3^	Size (bp)	Quail LG^4^	Chicken Chr:Mb^5^	E-value^6^
GCT1121	*ABCB6*	7	intron 9	113	CJA07	No hit^7^	-
GCT1333	*BCL2*	2	exon 1	237	CJA02	Chr2:67.684	2,00E-118
GCT1185	*CEBPD*	2	3' NC	184	CJA02	Chrun:70.609	8,50E-22
GCT1297	*CKB*	5	intron 2	529	CJA05	Chr5:47.115	1,70E-129
GCT1253	*COPS5*	2	exon 7	119	CJA02	Chrun:12.590	5,30E-27
GCT1168	*EYA1*	2	intron15	492	CJA02	Chr2:116.158	2,40E-174
GCT1820	*FASN*	18	3' NC	623	CJA18	Chr18:4.745	1,40E-229
GCT1237	*GATA6*	2	intron 4	170	CJA02	Chr2:101.861	9,60E-49
GCT1170	*GDAP1*	2	exon 3	110	CJA02	Chr2:117.706	2,40E-17
EXT0006	*MOS*	2	coding	468	CJA02	Chr2:110.417	1,90E-216
GCT1180	*PENK*	2	exon 2	314	CJA02	Chr2:110.572	1,60E-162
GCT1173	*PKIA*	2	3' NC	173	CJA02	Chr2:119.680	8,60E-65
EXT0007	*PRKDC*	2	intron12	786	CJA02	Chr2:107.114	2,20E-187
GCT1162	*RGS20*	2	3' NC	244	CJA02	Chr2:109.426	5,90E-78
GCT1233	*ROCK1*	2	intron30	179	CJA02	Chr2:101.378	3,30E-40
GCT1399	*STK29*	5	intron14	804	CJA05	Chr5:11.889	8,90E-247
GCT1401	*TYR*	1	Exon 2	188	CJA01	Chr1:179.594	3,30E-84
EXT0004	*ZFP161*	2	microsatellite	224	CJA02	Chr2:99.351	4,50E-81

^1^Corresponds to the gene name assigned by ICCARE to the chicken EST from which the primers were designed

^2^Determined by RH mapping [21].

^3^Determined by analogy to the orthologous human gene by ICCARE

^4^Linkage group

^5^Position of the quail fragment on the chicken genome sequence assembly, determined by BLASTN

^6^E-value of the quail-chicken alignment

^7^GCT1121 has a significant hit with chicken EST BI391579, that aligns with the chicken assembly at chrun:101609131–101609680

**Table 3:** FISH mapping quail small macrochromosomes and microchromosomes with chicken BAC clones

Chicken chromosome	BAC clones	Chicken genetic markers	Chicken genetic map (cM)	Quail chromosome or size estimate
6q11	bw27G19	LEI0192	31	6qcen
				
7p12	bw69P21	LEI0064	0	7qcen
7q13-q14	bw27C3	MCW0201	79	7qter
7q15-q16	bw26M16	ADL0169	165	7qter and 8qter^1^
7q16	P4G2	no	-	7q
				
8p12	bw29C17	MCW0275	6	8q
8q14	bw40L3	LEI0044	96	8qter
				
9	P1A6	GCT0016	41	9
9	bw14A21	MCW0134	132	9
				
10	bw8G10	MCW0132	88	10
				
11	bw35F15	LEI0143	0	11
11	bw29L10	MCW0097	18	11
				
13	bw14G12	MCW0244	0	8
13	bw35C23	MCW0104	74	8
				
14	bw43B20	MCW0296	0	11–14
14	bw32N20	MCW0225-*NTN2*	77	11–14
				
15	bw3B7	ADL0206	0	13–16
15	bw7G1	MCW0211	49	13–16
				
17	P5H12	no	-	16–17
				
18	bw19B13	MCW0045	0	16–18
18	bw1D2	MCW0219	47	16–18
				
19	P7E4	no	-	17–18
				
24	bw21H8	MCW0301	48	< 30
				
26	P2-7	GCT0906	67	21–24
				
27	bw9E8	MCW0146	35	23–25
				
28	bw36G5	LEI0135	0	17
28	bw32P6	ADL0299	60	17

^1^This BAC clone hybridizes to 1 chicken and 2 quail chromosomes
